# Clinical Features and Treatment Outcomes of COVID-19 Admissions in the Can Tho City Hospital of Tuberculosis and Respiratory Diseases, Vietnam: A Hospital-Based Observational Study

**DOI:** 10.3390/healthcare11111632

**Published:** 2023-06-02

**Authors:** Hung Do Tran, Tran Thanh Hung, Tran Hoang Thuy Phuong, Le Thanh Tam, Hung Gia Tran, Phuoc Huu Le

**Affiliations:** 1 Faculty of Nursing and Medical Technology, Can Tho University of Medicine and Pharmacy, 179 Nguyen Van Cu Street, Can Tho City 94000, Vietnam; 2 Faculty of Medicine, Can Tho University of Medicine and Pharmacy, 179 Nguyen Van Cu Street, Can Tho City 94000, Vietnam; tthung@ctump.edu.vn (T.T.H.); tghung@ctump.edu.vn (H.G.T.); 3 Can Tho City Hospital of Tuberculosis and Respiratory Diseases, Binh Hoa A Quarter, Phuoc Thoi Ward, O Mon District, Can Tho City 94000, Vietnam; thuyphuongtranhoang@gmail.com; 4 Can Tho University of Medicine and Pharmacy Hospital, 179 Nguyen Van Cu Street, Can Tho City 94000, Vietnam; lttam.bv@ctump.edu.vn; 5 Faculty of Basic Sciences, Can Tho University of Medicine and Pharmacy, 179 Nguyen Van Cu Street, Can Tho City 94000, Vietnam

**Keywords:** COVID-19, clinical characteristics, treatment outcomes, symptoms, mortality rate

## Abstract

Background: Coronavirus disease 2019 (COVID-19), caused by severe acute respiratory syndrome coronavirus 2 (SARS-CoV-2) infection, is among the top global health crises. As confirmed by the Vietnam Ministry of Health on 25th January 2023, Vietnam had a cumulative total of more than 11.52 million COVID-19 patients, including 10.61 million recoveries and 43,186 deaths. Objectives: This study aimed to describe the clinical and subclinical characteristics, treatment progress, and outcomes of 310 cases of SARS-CoV-2 infection. Methods: A total of 310 patients with medical records of SARS-CoV-2 were admitted to Can Tho City Hospital of Tuberculosis and Lung Diseases, Can Tho city, Vietnam, between July 2021 and December 2021. Demographic and clinical data, including laboratory examinations, of all the patients were collected and analyzed. Results: The median duration of hospital stay was 16.4 ± 5.3 days. There were 243 (78.4%) patients with clinical symptoms of COVID-19 and 67 (21.6%) patients without clinical symptoms. The common symptoms included cough (71.6% of 310 patients), fever (35.4%), shortness of breath (22.6%), sore throat (21.4%), loss of smell/taste (15.6%), and diarrhea (14.4%). Regarding treatment outcomes, 92.3% of the patients were discharged from the hospital, 1.9% of the patients suffered a more severe illness and were transferred to a higher-level hospital, and 5.8% of the patients died. The RT-PCR results were negative in 55.2% of the patients, and 37.1% of the patients had positive RT-PCR results with Ct values of >30 on the discharge/transfer day. Multivariate logistic regression analyses showed that comorbidity and decreased blood pH were statistically significantly related to the treatment outcomes of the patients with COVID-19 (*p* < 0.05). Conclusions: This study provides useful information (i.e., the clinical characteristics and treatment outcomes) on the COVID-19 pandemic in Vietnam during its biggest outbreak; the information may be used for reference and for making improvements in the handling of future health crises.

## 1. Introduction

Coronaviruses are a group of RNA viruses that usually cause mild-to-moderate upper respiratory tract illnesses in humans. However, three coronaviruses have caused more serious and fatal diseases in people: the SARS coronavirus (SARS-CoV, November 2002), which causes severe acute respiratory syndrome (SARS) [1]; the MERS coronavirus (MERS-CoV, in 2012), which causes Middle East respiratory syndrome (MERS) [2]; and SARS-CoV-2 in 2019, which causes coronavirus disease 2019 (COVID-19) [3]. In the past two decades, the SARS-CoV and MERS-CoV epidemics have caused a cumulative total of over 10,000 cases, with mortality rates of 10% and 37%, respectively [3]. The ongoing COVID-19 pandemic has become one of the most serious health crises in human history, spreading rapidly worldwide from January 2020 to the present [4,5]. According to the World Health Organization (WHO), since 24 January 2023, over 664 million confirmed cases and over 6.7 million deaths have been reported globally [6].

In Vietnam, the first COVID-19 infection was recorded on 22 January 2020 [7], and the country entered the fourth COVID-19 wave in late April 2021. There was an exponential increase in the number of cases in the fourth wave, mostly owing to the rapid spread of the delta and omicron variants [7,8]. As confirmed by the Vietnam Ministry of Health Vietnam on 25 January 2023, Vietnam had a cumulative total of more than 11.52 million COVID-19 patients, including 10.61 million recoveries and 43,186 deaths (with a confirmed case fatality rate of 0.37%) [9].

There have been studies on the clinical characteristics and treatment outcomes of COVID-19 patients around the world. Richardson et al. reported the presenting characteristics, comorbidities, and outcomes of 5700 hospitalized COVID-19 patients in the New York City area [10]. At a single US hospital, Kurihara et al. studied the clinical characteristics and outcomes of COVID-19 patients associated with acute respiratory distress syndrome (ARDS) and the characteristics and outcomes of those who underwent a lung transplant [11]. Wang et al. reviewed the medical records of 421 COVID-19 patients admitted to a mobile cabin hospital in Wuhan, China, in early 2020 [12]. Okogbenin et al. presented the clinical characteristics, treatment modalities, and outcomes of COVID-19 patients treated at an isolation and treatment center in Nigeria [13]. 

In Vietnam, earlier studies related to the COVID-19 pandemic were performed and published [5,14,15,16,17,18,19,20]. Indeed, the studies presented the factors associated with the duration of hospitalization among COVID-19 patients [14]; Vietnam’s experiences in combating the COVID-19 epidemic [15]; Vietnam’s policy responses to the COVID-19 pandemic [5]; the adaptive model of Vietnam’s health system organization to break SARS-CoV-2 transmission [16]; the effects of the COVID-19 pandemic on mental health [17]; the knowledge, attitudes, and practices of university students regarding COVID-19 [18]; COVID-19 waste treatment [19]; and the clinical features, isolation, and complete genome sequence of SARS-CoV-2 from the first two patients in Vietnam [20]. However, to the best of our knowledge, a full study of the clinical and subclinical features and treatment outcomes of COVID-19 patients admitted to a representative hospital in Vietnam has not yet been reported.

Although the clinical characteristics, treatment outcomes, and other associated factors among COVID-19 patients have been reported in some studies [10,11,12,13], the reported results varied greatly among the studies owing to the marked variation in patient demographics, access to healthcare, the healthcare infrastructure, and the preparedness of the regions [21,22]. Therefore, a study on the clinical characteristics and treatment outcomes among Vietnamese patients with a SARS-CoV-2 infection admitted to a typical/representative hospital in Vietnam will provide useful information for the making of health policies, not only for the current pandemic but also for future global events. In this study, we present the demographic, clinical, and subclinical characteristics; the admission and respiratory status; the treatment progress; and the outcomes of 310 COVID-19 patients admitted to Can Tho City Hospital of Tuberculosis and Lung Diseases, Can Tho city, Vietnam, between July 2021 and December 2021. 

## 2. Materials and Methods

### 2.1. Patients and Data Collection

The study population included 310 patients aged ≥18 years with laboratory-confirmed COVID-19 who were admitted to Can Tho City Hospital of Tuberculosis and Respiratory Diseases, Vietnam, between July 2021 and December 2021. We used positive reverse transcriptase polymerase chain reaction (RT-PCR) results to confirm SARS-CoV-2 infection, and we excluded patients with lung diseases caused by other viruses, bacteria, and fungi. 

The total number of COVID-19 patients admitted to Can Tho City Hospital of Tuberculosis and Respiratory Diseases, Vietnam, for treatment between July and December 2021 was 979, and the required sample size was 310; thus, the sampling distance was k = 979/310 = 3.2. Here, we chose k = 2 to create a list of confirmed COVID-19 patients (from cases 1 to 310) treated between July and December 2021. Well-trained and experienced investigators viewed and collected the medical records (i.e., the clinical and laboratory information, chest X-ray results, etc.) and filled out the questionnaires.

It is worth noting that the hospital in this study is one of the major treatment centers of Can Tho city, which has a population of ~1.3 million. As of 25 January 2023, Can Tho city had had 76,570 confirmed COVID-19 cases and 598 deaths [9]. The data collection period was selected because it belongs to the fourth wave of the pandemic, during which there were exploding COVID-19 case numbers across Vietnam as well as in Can Tho city [9]. This study was approved by the Can Tho University of Medicine and Pharmacy Ethics Committee.

The clinical and laboratory progress of the patients was obtained by reading the chest X-ray results and by the filling out of the questionnaires by the medical doctors. The questionnaire included patient data, such as their demographics, presenting signs and symptoms, clinical and subclinical characteristics, drug use, length of hospitalization, duration of drug treatment, and the time between positive and negative test results. 

### 2.2. The Scale of the Disease Severity and Clinical Progress Assessment

The severity of COVID-19 was classified according to Decision No. 3416/QD-BYT, dated 14th July 2021, of the Ministry of Health of Vietnam on Guidance on Diagnosis and Treatment of Acute Respiratory Infection Caused by the New Strain of Corona Virus (2019-nCoV) [23]. The disease severity and clinical progress assessment are described in Table 1 and Table 2.

### 2.3. Statistical Analysis

The collected data were analyzed using SPSS Statistics for Windows, version 20.0. The data are presented as frequencies and percentages for the qualitative variables and as mean and standard deviation (if normally distributed) for the quantitative variables. Pearson’s chi-square test was used for the categorical variables, and Fisher’s exact test was applied in cases where the frequency of one of the analyzed groups was less than 5. For quantitative variables with a normal distribution, Student’s *t*-test was used for statistical analysis. Stepwise multivariable logistic analysis was conducted to identify variables that could potentially impact the outcome under investigation over time. The relationships between the factors and treatment outcomes are presented as odds ratios (ORs) and 95% confidence intervals (CIs). The relationship was considered statistically significant when the *p*-value was <0.05. The categorical variables are presented as frequencies and percentages, whereas the quantitative variables are presented as means and standard deviations in the case that they were normally distributed; otherwise, they are shown as medians and interquartile ranges (IQRs).

## 3. Results

### 3.1. COVID-19 Patients’ Demographic and Clinical Characteristics 

Of the 310 study subjects, 134 (43.2%) were male, whereas 176 (56.8%) were female. There were 10 (3.2%) patients aged <20 years; 68 (21.9%) patients aged 20–39 years; 139 (44.8%) patients aged 40–59 years; and 93 (30%) patients aged ≥60 years (Table 3). There were 243 (78.4%) patients with clinical symptoms and 67 (21.6%) without clinical symptoms. The common symptoms included cough (174; 71.6%), fever (86; 35.4%), shortness of breath (55; 22.6%), sore throat (52; 21.4%), loss of smell/taste (38; 15.6%), diarrhea (35; 14.4%), muscle pain (14; 5.8%), nausea (8; 3.3%), and other symptoms (16; 6.6%). More than half of the patients (168; 54.2%) had no underlying disease. Among the patients with comorbidities (142; 45.8%), the proportions of patients with one, two, and more than two comorbidities were 29.7%, 12.9%, and 3.2%, respectively (Table 3). Among 142 (45.8%) patients with at least one underlying disease, the most common conditions/diseases were hypertension (88; 62.0%); diabetes (51; 35.9%); tuberculosis under ongoing treatment (12; 8.5%); obesity (9; 6.3%); tuberculosis with completed treatment (8; 5.6%); chronic obstructive pulmonary disease (COPD; 6; 4.2%); brain stroke in the past (5; 3.5%); and other diseases (3; 3.5%). 

### 3.2. Admission Status of the COVID-19 Patients

At admission, 9.7% of the COVID-19 patients had hypertension, which was defined as a systolic blood pressure of ≥140 mmHg and/or a diastolic blood pressure of ≥90 mmHg; 280 (90.3%) patients had normal blood pressure. Regarding the oxygen saturation level (SpO_2_) condition of the patients, 11.9% of the patients had SpO_2_ < 94%, 15.2% of the patients had SpO_2_ in the range of 94–96%, and 72.9% had SpO_2_ > 96% (Table 4).

### 3.3. Respiratory Status of the COVID-19 Patients

SARS-CoV-2 can cause acute respiratory distress syndrome; thus, oxygen therapy is prudent for patients with SARS-CoV-2 at different stages of the disease. The choice of the oxygen delivery device depends on the availability and on the patient’s status. Of 310 patients, 239 (77.1%) did not require further oxygen support (they breathed room air normally); 33 (10.6%) received oxygen via nasal cannula (1–4 L/min); 24 (7.7%) breathed oxygen via bag-mask ventilation (5–10 L/min); 8 (2.6%) required a ventilator (10–15 L/min); and 6 (1.9%) had to receive HFNC (40–60 L/min) (Table 5). 

### 3.4. Subclinical Conditions of COVID-19 Patients 

Of the patients, 30.6% had anemia, 0.3% had leukopenia, and 26.5% had leukocytosis. We found that 6.8% of the patients presented a decrease in neutrophil counts (<4 × 10^−3^/mL); 18.4% of the patients had an increase in neutrophil counts (>7 × 10^−3^/mL); 40% of the patients showed a decrease in lymphocyte counts (<2 × 10^−3^/mL); and 7.1% of the patients exhibited an increase in lymphocyte counts (>3 × 10^−3^/mL). Of the patients, 6.8% had decreased blood pH (<7.35), and 5.5% had increased blood pH (>7.45). The proportion of patients with decreased arterial P_O2_ (P_CO2_) was 17.4% (5.5%) and that of the patients with increased arterial P_O2_ (P_CO2_) was 1.6% (8.7%). Of the patients, 32.6% had increased D-dimer (≥0.5 µg/mL); 31.6% had an increased aspartate aminotransferase (AST) level (>34 U/L); and 37.4% had an increased alanine aminotransferase (ALT) level (>55 U/L). Of the patients, 7.4% had hypouricemia; 14.8% had hyperuricemia; 1.9% had decreased creatinine (<44 µmol/L); and 11.6% had increased creatinine (>110 µmol/L). 

Regarding the results of the straight chest X-ray, of the 310 COVID-19 patients, 133 (42.9%) had normal results, while 177 (57.1%) showed disorders on the chest radiography, including focal opacity (25.5%), diffuse opacity (15.5%), solidified lesions (11.3%), and diffuse solidified lesions (4.8%). Signs of ground-glass opacity occur when a patient experiences lung damage caused by SARS-CoV-2. This image usually appears predominantly in the periphery of both lungs. In our study, opacities accounted for the highest percentage, indicating that most of the patients were in the acute stage. 

To prevent blood clotting disorders, 39% of the COVID-19 patients who had an increased D-dimer level were prescribed enoxaparin at a dose of 1 mg/kg daily or heparin at a typical dose of 5000 units per 12 h via intravenous injection [24]. Remdesivir was prescribed to 31.9% of the patients [25], of whom 88.9% had no side effects.

### 3.5. Clinical Progress during the Treatment of COVID-19 Patients

**Figure 1:**
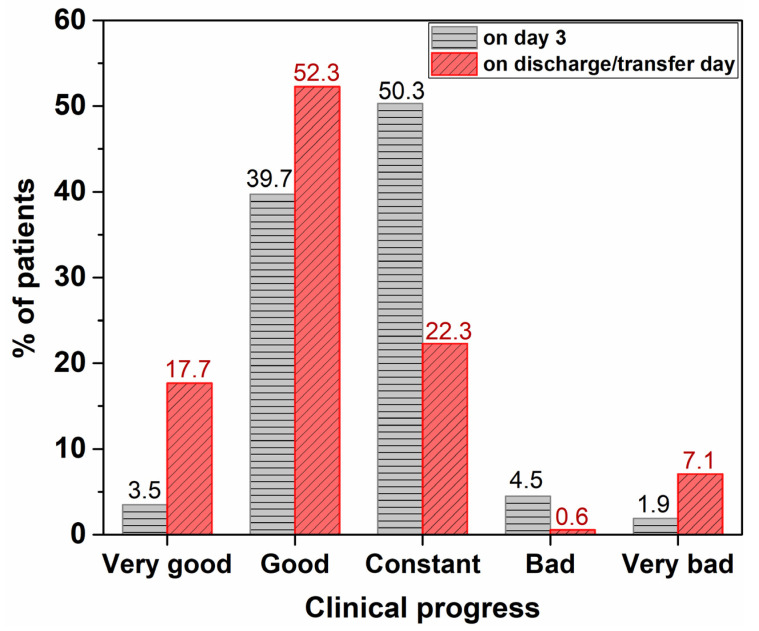
Clinical progress of COVID-19 patients after treatment on day 3 and discharge/transfer day. A detailed description of the clinical progress scale is presented in Table 2.

The treatment of COVID-19 patients followed the Guidance of the Ministry of Health of Vietnam, according to Decision No. 3416/QD-BYT, issued on 14 July 2021 [23]. Figure 1 shows the clinical progress of the patients on day 3 and on the discharge/transfer day. The clinical progress scale included five levels, namely, “very good”, “good”, “constant”, “bad”, and “very bad” (see Table 2 for detailed description of the scale). This study found that 3.5% of the patients achieved very good progress on day 3, and this percentage increased to 17.7% on the discharge/transfer day (Figure 1). In addition, 39.7% of the patients obtained good progress on day 3, and the good progress percentage increased up to 52.3% at the end of the treatment. Half of the patients (50.3%) presented a constant status after 3 days of treatment, and the percentage of this patient group reduced to 22.3% on the discharge/transfer day (Figure 1). However, there were small percentages of patients with bad progress (4.5%) and very bad progress (1.9%) on day 3; these numbers changed to 0.6% with bad progress and 7.1% with very bad progress on the discharge/transfer day (Figure 1). 

### 3.6. The Treatment Results of the COVID-19 Patients 

The mean hospital stay was 16.4 ± 5.3 days, while the shortest stay was 1 day, and the longest stay was 44 days (Table 6). 

Our study found that 286 (92.3%) patients recovered and were discharged from the hospital, 6 (1.9%) worsened and were transferred to a higher-level hospital, and 18 (5.8%) died. In addition, 171 (55.2%) patients had negative RT-PCR results, but the RT-PCR results of 115 (37.1%) patients remained positive (Ct value > 30) on the discharge/transfer day. 

### 3.7. Factors Associated with the Treatment Outcomes for the COVID-19 Patients 

The patients were divided into groups according to whether their treatment outcomes were successful or unsuccessful. The successful treatment group included patients who recovered and were discharged from the hospital, while the unsuccessful treatment group included patients who had clinically worsened, those who were transferred to a higher-level hospital, and those who had died. Table 7 presents the primary characteristics of the COVID-19 patients according to the treatment outcomes. The COVID-19 patients without comorbidities had a statistically significantly higher rate of successful treatment than those with comorbidities (OR = 9.5; 95% CI of 2.8–32.7; *p* < 0.001). This is because comorbidities can affect the pathophysiology of COVID-19 patients and cause the worsening of the COVID-19 infection. In addition, the patients who breathed room air had a higher rate of successful treatment than the patient group with oxygen support (99.6% vs. 67.6%). This difference was statistically significant, with OR = 114.04, 95% CI = 15.0–864.9, and *p* < 0.001. Moreover, the patients with a normal hemoglobin level had a higher treatment success rate (93.5%) than the patients with anemia (89.5%). However, this difference was not statistically significant.

Patients with normal white blood cell (WBC) counts had a higher rate of successful treatment than the patients with abnormal WBC counts (96.9% vs. 79.5%), and this difference was statistically significant (OR = 8.1, 95% CI of 3.2–20.4, and *p* < 0.001). The COVID-19 patients with normal AST levels had a significantly higher rate of successful treatment than did those with elevated AST levels (94.8% vs. 86.7%; OR = 2.8, 95% CI of 1.2–6.5; *p* = 0.013). Similarly, the normal ALT level group had a higher treatment success rate (95.4%) than the elevated ALT level group (87.1%), and this difference was statistically significant, with OR = 3.1, 95% CI of 1.3–7.2, and *p* = 0.008. In addition, the normal blood urea patient group had a higher treatment success percentage than the patient group with an abnormal blood urea index (95.9% vs. 79.7%), and this finding was statistically significant (OR = 5.9; 95% CI of 2.5–14.0; *p* < 0.001). Moreover, the patients with a normal blood urea index had a higher treatment success rate than the group of patients with an abnormal blood urea index. The difference was statistically significant, with OR = 5.9, 95% CI = 2.5–14.0, and *p* < 0.001. In addition, creatinine level was also a factor associated with treatment outcome, and the normal creatinine patient group had a higher treatment success rate than the abnormal creatinine patient group (OR = 4.6; 95% CI of 1.9–11.3; *p* = 0.002, obtained via Fisher’s exact test). Furthermore, the patients with normal blood pH had a significantly higher treatment success rate (97.4%) than those in the patient group with increased blood pH (70.6%; OR = 15.8; *p* < 0.001) and the patient group with decreased blood pH (42.9%; OR = 50.5; *p* < 0.001). 

The COVID-19 patient group with normal chest X-ray results had a treatment success rate of 100%, while the group with abnormal results had a lower treatment success rate of 78.6%, and this difference was statistically significant (*p* < 0.001). A higher treatment success rate was observed for the patient group with normal D-dimer levels (>1000 ng/mL) than for the patient group with an increased D-dimer level (>1000 ng/mL) (99.5% vs. 77.2%); this difference was statistically significant (OR = 61.3 and *p* < 0.001). 

The patients who did not use remdesivir had a higher treatment success rate than those who did (96.7% vs. 82.8%). This difference was statistically significant (OR = 6.0 and *p* < 0.001). In the patient group using remdesivir (*n* = 99, 31.9%), 76 (86.4%) had successful outcomes and 12 (13.6%) had unsuccessful outcomes after treatment with remdesivir for 6–10 days; the rates of successful and unsuccessful outcomes were 6 (54.5%) and 5 (45.5%) after treatment with remdesivir for 5 days, respectively. This means that the group using remdesivir for 6–10 days had a higher success rate than the group using remdesivir for 5 days, and the difference was statistically significant (OR = 5.3 and *p* = 0.02). 

Using multivariate logistic regression analysis, it was found that of the factors listed in Table 8 comorbidity and decreased blood pH were the two statistically significant factors related to the treatment outcomes of COVID-19 patients, with *p*-values of 0.045 and 0.018, respectively. 

## 4. Discussion

Regarding the population of this study (*n* = 310), the percentage of female patients (58.6%) was slightly higher than that of the male patients (43.2%); this was similar to the populations (*n* = 133) comprising 51.1% female and 48.9% male patients in [26] and 52.3% female and 47.7% male patients in [27]. However, the populations of some early COVID-19 studies had more male patients than female patients, involving ratios such as male/female = 52%/48% [28,29]; male/female = 50.8%/49.2% [12]; male/female = 60%/40% [30]; male/female = 65.9%/34.1% [31]; and male/female = 51.8%/48.2% [22]. The male:female ratio was up to 2.7:1 for an early study published in January 2020 [4]. 

Regarding age group characteristics, the age groups of the COVID-19 patients were 40–59 years old (44.8%), ≥60 years old (30%), 20–39 years old (21.9%), and <20 years old (3.2%). This suggests that COVID-19 has a high prevalence in the middle-aged and elderly population but a lower prevalence in young people (especially those <20 years old).

Among the 243 (78.4%) patients with clinical symptoms, nearly three fourths (71.6%) had a cough. This was followed by fever (35.4%), dyspnea (22.6%), sore throat (21.4%), loss of smell/taste (15.6%), diarrhea (14.4%), muscle pain (5.8%), nausea (3.3%), and other symptoms (6.6%). The symptoms seen in COVID-19 patients are mostly nonspecific respiratory symptoms, which can be seen in many respiratory infections of other etiologies. Most studies published between 2020 and early 2021 found that the two most common symptoms were fever followed by cough: fever (98%) and cough (76%) [3]; fever (88.5%) and cough (68.6%) [30]; fever (85.3%) and cough (52.6%) [32]; fever (98%) and cough (76%) [4]; fever (60.6%) and cough (52.0%) [12]; and fever (60.66%) and cough (52.46%) [33]. In contrast to previous reports, our study, which was conducted from July 2021 to December 2021, recorded that the cough percentage (71.6%) was higher than that of fever (35.4%) as the most common symptom. Okogbenin et al. [13] also observed that the two most commonly presented symptoms included cough (34.0%) and fever (30.3%). During the pandemic period from July 2021 to December 2021, most hospitals in the city were overloaded, and COVID-19 patients were often treated at home before they were hospitalized with clear symptoms. In addition to lung damage, COVID-19 patients usually suffer from damage to the upper respiratory tract mucosa at both the macroscopic and microscopic levels, which causes the persistent dry cough [34].

At hospital admission, the proportion of COVID-19 patients with hypertension was 9.7%, which was lower than that of 27% in ref. [35] and 15% in ref. [3]. Moreover, patients with SpO_2_ < 94%, SpO_2_ in the range of 94–96%, and SpO_2_ > 96% accounted for 11.9%, 15.2%, and 72.9%, respectively (Table 4). A lower SpO_2_ level is an early indicator that the patient requires more medical attention [36,37].

Regarding the patients’ respiratory status, the majority of patients (77.1%) breathed room air normally, while the other patients had to have a nasal cannula (10.6%), bag-mask ventilation (7.7%), a ventilator (2.6%), or HFNC (1.9%). Different oxygen delivery devices were used appropriately, depending on the patient’s status and the availability of the devices. 

As shown in Figure 1, the treatment generally showed good effectiveness and positive progress over the treatment period. Indeed, the proportion of patients with “very good” and “good” outcomes was 43.2% on day 3 and 70% on the discharge/transfer day. However, there remained a small proportion of patients with “bad” and “very bad” results of 6.4% on day 3 and 7.7% on discharge/transfer day.

The percentages of COVID-19 patients with anemia, leukocytosis, and leukopenia were 30.6%, 26.5%, and 0.3%, respectively. Typically, the normal ranges of neutrophil and lymphocyte counts were 1.7 × 10^9^–7.7 × 10^9^ L^−1^ and 0.4 × 10^9^–4.4 × 10^9^ L^−1^, respectively. Neutrophils are the most abundant type of white blood cell (WBC), accounting for 55–70% of WBCs. Lymphocytes, a type of WBC, normally account for 25–40% of WBCs. In this study, 6.8% of the patients had a decrease in neutrophils (<55% of WBCs); 18.4% of the patients had an increase in neutrophils (>70% of WBCs); and 74.8% of the patients had neutrophils in the normal range. In addition, 52.9% of the patients had a normal percentage of lymphocytes (25–40% of WBCs), 40% of the patients had a decrease in lymphocyte percentage (<25% of WBCs), and 7.1% of the patients had an increase in lymphocyte percentage (>40% of WBCs). Zhu et al. found that the white blood cell (WBC) count at admission was significantly associated with mortality in COVID-19 patients, and it was recommended that the patients with a higher level of WBCs should be given more attention in the treatment [27]. 

D-dimer is a fibrin degradation product released upon the cleavage of cross-linked fibrin by plasmin [38]. Of the 310 COVID-19 patients, 121 (39%) had D-dimer elevation (≥0.50 mg/mL), which was lower than the 80.1% reported in ref. [38]. Recent studies have linked COVID-19 to hemostatic abnormalities; these studies include an observational study of elevated D-dimer levels [38]. Indeed, an increase in D-dimer levels correlates with disease severity and outcome [39]. A retrospective cohort study of 191 patients showed that D-dimer levels > 1.0 μg/mL (*p* = 0.0033) were associated with increased mortality in COVID-19 patients [40]. The authors found that a D-dimer level of >2.0 μg/mL on admission was the optimal threshold for predicting mortality in COVID-19 patients [40]. Furthermore, Huang et al. found that elevated D-dimer concentrations on admission could be used to place patients in critical care [3]. The results of these studies suggest that D-dimer levels can be used as a prognostic marker and can help clinicians monitor those who are likely to deteriorate earlier [41]. 

To prevent blood clotting disorders, 39% of the patients who had an increased D-dimer level were prescribed enoxaparin at a dose of 1 mg/kg daily or heparin at a typical dose of 5000 units per 12 h by intravenous injection, strictly following the Guidelines of the Ministry of Health of Vietnam [23]. However, it remains unclear how enoxaparin and heparin drugs influence the prognosis and progression of COVID-19. According to the literature, the use of anticoagulation therapy (e.g., mainly low molecular weight heparin) in patients with coagulopathy or a marked rise in D-dimer levels in the setting of COVID-19 was found to be associated with better prognosis in severe cases [41,42]. In addition, according to Ning Tang et al. [42], no difference in the 28-day mortality was found between heparin users and non-users (30.3% vs. 29.7%, *p* = 0.910).

The number of patients assigned to the remdesivir group was 99 (31.9%). Among them, 88.9% of the patients had no side effects after using remdesivir, but 3% of the patients had hypersensitivity reactions, and 8.1% had elevated liver enzymes. Consequently, 3% of the patients were advised by the doctor to stop using remdesivir, and 97% of the patients used the full dose of the drug. 

Regarding treatment outcomes, 286 (92.3%) patients were discharged from the hospital. Among the discharged patients, 55.2% had negative RT-PCR results and 37.1% had positive PCR results (Ct value > 30). The median duration of stay in the hospital was 16.4 ± 5.3 days, which was shorter than the median lengths of hospital stay of 24 days [28], 21 days [14], and 21 days for patients with comorbidities and 14 days for patients without comorbidities [43]. In addition, it was slightly longer than the median hospital stay of 13 days reported in ref. [44]. A total of 1.9% of the patients with more severe disease was transferred to a higher-level hospital. The mortality rate in this study was 5.8%, which was comparable to that of 5.6% in a review article [22], but higher than that of 3.1% in a study in Indonesia [45]. The mortality rates were found to depend on age. For patients aged <50 years, the mortality rates were 5% in Jakarta (Indonesia) [28], 5% in New York City (USA) [10], and 4% in the UK [46], but the rates for elderly patients (≥50 years) were 21% in Jakarta, 27% in the US [10], and 29% in the UK [46]. Older age has been consistently associated with severe disease; thus, the mortality rate in the elderly patient group was significantly higher than that in the younger patient group [10,28,46]. 

We conducted univariate and multivariate logistic regression analyses of the factors related to the treatment outcomes of COVID-19 patients. The results of the univariate logistic regression analyses yielded 11 risk factors: comorbidity, breathing room air, normal WBCs, normal AST level, normal ALT level, normal blood urea, normal creatine, normal chest X-ray, normal D-dimer, decreased blood pH, and no remdesivir use; these were independently related to treatment outcome in the study population (*p* < 0.05). Meanwhile, the multivariate logistic regression analyses found that comorbidity and decreased blood pH were the only two factors significantly related to the treatment outcomes of the COVID-19 patients (*p* < 0.05). E.M. Wardani et al. found that patients with comorbidities had a significantly longer average length of stay than those without comorbidities (21 vs. 14 days) [43]. M. Kieninger et al. found that lower blood pH was a strong prognostic factor for fatal outcomes in critically ill COVID-19 patients at an intensive care unit [47]. 

### Limitations

This hospital-based observational study needs to generate a large amount of missing data. For instance, some characteristics of the patients (e.g., smoking, alcohol use, vaccination status, etc.) were not collected. In addition, this study was limited by the relatively small sample size and selection bias as it was conducted at one hospital in a single country and did not include longitudinal data. In this study, we attempted to reduce possible bias by assigning only a few well-trained and experienced medical doctors to collect the data and by performing multivariate analysis. 

## 5. Conclusions

This study reported on the demographics, clinical and subclinical characteristics, admission and respiratory status, treatment progress, and outcomes of 310 COVID-19 patients admitted to the Can Tho City Hospital of Tuberculosis and Lung Diseases, Can Tho city, Vietnam, between July 2021 and December 2021. There were 243 (78.4%) patients with clinical symptoms and 67 (21.6%) without clinical symptoms of COVID-19. The common symptoms included cough, fever, shortness of breath, sore throat, loss of smell/taste, and diarrhea. Patients with SpO_2_ > 96%, SpO_2_ in the range of 94–96%, and SpO_2_ < 94% accounted for 72.9%, 15.2%, and 11.9%, respectively. The majority of patients (77.1%) breathed room air normally; some of the patients had to have a nasal cannula (10.6%), bag-mask ventilation (7.7%), a ventilator (2.6%), and HFNC (1.9%). D-dimer elevation was observed in 39% of the patients. The treatments generally showed good effectiveness and positive progress over the treatment period. Indeed, the proportion of patients with “very good” and “good” outcomes accounted for 43.2% on day 3 and 70% on the day of discharge/transfer. The mean hospital stay duration was 16.4 ± 5.3 days. Of the COVID-19 patients, 92.3% were discharged from the hospital, 1.9% suffered a more severe illness and were transferred to a higher-level hospital, and 5.8% died. On the discharge/transfer day, 55.2% of the patients had negative RT-PCR results and 37.1% of the patients still had positive RT-PCR results (Ct value > 30). The multivariate logistic regression analyses found that comorbidity and decreased blood pH were the only two factors significantly related to the treatment outcomes of patients with COVID-19 (*p* < 0.05). This study reported the clinical characteristics and treatment outcomes of a hospital in Vietnam and provides useful information on the COVID-19 pandemic in Vietnam during its biggest outbreak; this information is for reference and for making improvements in the handling of future health crises.

## Figures and Tables

**Table 1:** The scale of the disease severity.

Asymptomatic/Pre-symptomatic	- Positive for SARS-CoV-2 using a test but no symptoms that are consistent with COVID-19. - Breathing rate < 20 breaths/min, SpO_2_ > 96% on room air.
Mild Illness	- Patients with COVID-19 have non-specific clinical symptoms, such as fever, dry cough, sore throat, nasal congestion, fatigue, headache, muscle aches, loss of taste, smell, diarrhea, etc. - Breathing rate < 20 breaths/min, SpO_2_ > 96% on room air. - When awake, the patient can function autonomously. - Chest X-ray is normal or shows only minimal damage.
Moderate Illness	- General condition: the patient has non-specific clinical symptoms. - Respiratory: there are signs of pneumonia with dyspnea, rapid breathing 20–25 times/min, lung crackles, and no signs of severe respiratory failure, with SpO_2_ of 94–96% on room air. The person may have difficulty breathing during exertion (walking around the house, going upstairs). - Circulation: fast or slow pulse, dry skin, tachycardia, normal blood pressure. - Consciousness: awake. - Chest X-ray and chest computed tomography (CT): there are lesions; the lesions cover < 50%. - Ultrasound: B-wave image. - Arterial blood gas: PaO_2_/FiO_2_ > 300 mmHg.
Severe Illness	- Respiratory: signs of pneumonia accompanied by any one of the following signs: respiratory rate > 25 breaths/min, severe shortness of breath, contraction of accessory respiratory muscles, SpO_2_ < 94% on room air. - Circulatory: tachycardia or possibly bradycardia, blood pressure normal or elevated. - Nervous: the patient may be restless or lethargic and tired.
Critical Illness	- Respiratory: tachypnea > 30 breaths/min or <10 breaths/min, severe acute respiratory distress syndrome with labored breathing, abnormal breathing or need for respiratory support with a high-flow nasal cannula (HFNC), continuous positive airway pressure (CPAP), or mechanical ventilation. - Nervous: decreased consciousness or coma. - Circulation: tachycardia, possibly bradycardia, low blood pressure. - Kidney: little urine or anuria. - Chest X-ray and chest CT: there are lesions; lesions cover more than 50%. - Arterial blood gas: PaO_2_/FiO_2_ < 200 mmHg, respiratory acidosis, blood lactate > 2 mmol/L. - Ultrasound: many B-wave images.

**Table 2:** The description of the clinical progress scale in this study.

Clinical Progress	Description
Very good	COVID-19 patients who had significantly improved general, clinical, and subclinical conditions as compared to those conditions at the time of admission (e.g., increased SpO_2_ > 96% and no required respiratory intervention).
Good	COVID-19 patients who had noticeably improved general, clinical, and subclinical conditions as compared to those conditions at the time of admission (e.g., increased SpO_2_).
Constant	COVID-19 patients who had almost the same general, clinical, and subclinical conditions as compared to those conditions at the time of admission. Typically, the patients remained in almost the same SpO_2_ condition and had the same need for respiratory intervention.
Bad	COVID-19 patients who had general, clinical, and subclinical conditions of severe illness after treatment. The typical classification criterion was noticeably decreased SpO_2_ as compared to the SpO_2_ at the time of admission.
Very bad	COVID-19 patients who had general, clinical, and subclinical conditions of critical illness after treatment. Some typical classification criteria were decreased SpO_2_, triglyceride disorders, hemodynamic disturbances, or cytokine storm.

**Table 3:** Baseline characteristics of the patients with COVID-19.

Variables	Frequency (*n*)	Ratio (%)
Gender		
Male	134	43.2%
Female	176	56.8%
Age (years)		
<20	10	3.2
20–39	68	21.9
40–59	139	44.8
≥60	93	30.0
Clinical symptoms of COVID-19		
Yes	243	78.4
No	67	21.6
Clinical symptoms appearing in the patient (*n* = 243)		
Cough	174	71.6
Fever	86	35.4
Sore throat	52	21.4
Loss of smell/taste	38	15.6
Nausea	8	3.3
Diarrhea	35	14.4
Shortness of breath	55	22.6
Muscle pain	14	5.8
Other	16	6.6
Number of comorbidities (disease(s))		
0	168	54.2
1	92	29.7
2	40	12.9
>2	10	3.2

**Table 4:** Blood pressure and SpO_2_ status of the COVID-19 patients at the time of admission.

Variables	Frequency (*n*)	Ratio (%)
Blood pressure		
Normal	280	90.3
Hypertension	30	9.7
SpO_2_		
<94%	37	11.9
94–96%	47	15.2
>96%	226	72.9

**Table 5:** Respiratory support for COVID-19 patients.

Respiratory Support	Frequency (*n*)	Ratio (%)
Breathed room air	239	77.1
Nasal cannula	33	10.6
Bag-mask ventilation	24	7.7
Ventilator	8	2.6
High-flow nasal cannula (HFNC)	6	1.9
Total	310	100

**Table 6:** Length of hospital stay of COVID-19 patients.

Length of Hospital Stay	Frequency (*n*)	Ratio (%)
<10 days	20	6.5
10–14 days	64	20.6
>14 days	226	72.9
Total	310	100
Minimum length of hospital stay (day)	1	
Maximum length of hospital stay (day)	44	
Mean ± standard deviation (day)	16.4 ± 5.3	

**Table 7:** Main characteristics of COVID-19 patients according to treatment outcomes.

Variable	Treatment Outcome		OR (95% CI)	*p*-Value
	Successful *n* (%)	Unsuccessful *n* (%)		
**Comorbidity**				
No	165 (98.2%)	3 (1.8%)	9.5 (2.8–32.7)	<0.001
Yes	121 (85.2%)	21 (14.8%)		
**Respiratory condition**				
Breathed room air	238 (99.6 %)	1 (0.4%)	114.04 (15.0–864.9)	<0.001
Oxygen support	48 (67.6%)	23 (32.4%)		
**Hemoglobin**				
Normal (male 14–16 g/dL; female 12.5–14.5 g/dL)	201 (93.5%)	14 (6.5%)	1.7 (0.7–4.0)	0.223
Anemia (male < 13 g/dL; female < 12 g/dL)	85 (89.5%)	10 (10.5%)		
**White blood cells**				
Normal (4 × 10^9^–10 × 10^9^/L)	220 (96.9%)	7 (3.1%)	8.1 (3.2–20.4)	<0.001
Abnormal (<4 × 10^9^ /L or >10 × 10^9^/L)	66 (79.5%)	17 (20.5%)		
**Aspartate aminotransferase (AST) level**				
Normal (≤37 U/L)	201 (94.8%)	11 (5.2%)	2.8 (1.2–6.5)	0.013
Increased (>37 U/L)	85 (86.7%)	13 (13.3%)		
**Alanine aminotransferase (ALT) level**				
Normal (≤40 U/L)	185 (95.4%)	9 (4.6%)	3.1 (1.3–7.2)	0.008
Increased (>40 U/L)	101 (87.1%)	15 (12.9%)		
**Blood urea**				
Normal (2.5–7.5 mmol/L)	231 (95.9%)	10 (4.1%)	5.9 (2.5–14.0)	<0.001
Abnormal (<2.5 or >7.5 mmol/L)	55 (79.7%)	14 (20.3%)		
**Creatinine**				
Normal (male 62–120 µmol/L; female 53–100 µmol/L)	253 (94.4%)	15 (5.6%)	4.6 (1.9–11.3)	0.002 *
Abnormal (male < 62 or >120 µmol/L; female < 53 or >100 µmol/L)	33 (78.6%)	9 (21.4%)		
**Blood pH**				
Normal (7.37–7.45)	265 (97.4%)	7 (2.6%)	-	-
Increased (>7.45)	12 (70.6%)	5 (29.4%)	15.8 (4.4–57.0)	<0.001
Decreased (<7.37)	9 (42.9%)	12 (57.1%)	50.5 (16.1–158.6)	<0.001
**Chest X-rays**				
Normal	133 (100%)	0 (0%)	42.6 (2.6–707.6)	<0.01
Abnormal	153 (86.4%)	24 (13.6%)		
**D-dimer**				
Normal (<1000 ng/mL)	208 (99.5%)	1 (0.5%)	61.3 (8.1–461.9)	<0.001
Increased (>1000 ng/mL)	75 (77.2%)	23 (22.8%)		
**Remdesivir use**				
No	204 (96.7%)	7 (3.3%)	6.0 (2.4–15.1)	<0.001
Yes	82 (82.8%)	17 (17.2%)		

OR = odds ratio; CI = confidence interval; * Fisher’s Exact Test.

**Table 8:** Multivariate logistic regression analyses of factors related to the treatment outcomes of COVID-19 patients.

Factor	OR (95% CI)	*p*-Value
Comorbidity	4.9 (1.0–23.3)	0.045
Breathed room air	-	0.998
Normal white blood cells	2.5 (0.7–8.8)	0.153
Normal AST level	2.0 (0.6–7.1)	0.281
Normal ALT level	2.2 (0.6–7.8)	0.214
Normal blood urea	1.8 (0.4–7.6)	0.439
Normal creatinine	2.7 (0.5–13.8)	0.238
Normal D-dimer	-	0.998
Decreased blood pH	5.8 (1.4–24.7)	0.018
Remdesivir not used	0.6 (0.2–2.6)	0.530

OR = odds ratio; CI = confidence interval.

## Data Availability

The data presented in this study are available from the first and corresponding authors upon reasonable request.
